# The Effects of Wages and Training on Intent to Switch or Leave Among Direct Care Workers

**DOI:** 10.1093/geroni/igac035

**Published:** 2022-05-20

**Authors:** Kensaku Kishida

**Affiliations:** Graduate School of Humanities and Social Sciences, Okayama University, Okayama, Japan

**Keywords:** Long-term care, Turnover, Workforce issues

## Abstract

**Background and Objectives:**

Although most studies have not separated turnover of direct care workers (DCWs) into those who switch to another organization (switchers) and those who leave the industry (leavers), switchers and leavers have different impacts on the facilities they quit and the labor market for DCWs. We distinguished between intent to switch and intent to leave and investigated the impact of wages and training on each turnover intention.

**Research Design and Methods:**

Data were obtained from Japan’s Fact-Finding Survey on Long-term Care Work. We included DCWs (*n* = 7,311) in the analyses and used multinomial regression by sex and provider type to compare those who wanted to switch and those who wanted to leave with those who wanted to remain in their current workplace.

**Results:**

The impacts of an increase in wages and a higher training score were larger for intent to switch than intent to leave. Compared with wages, the impact of training was greater. The impact of job characteristics on turnover intention varied between women and men and across provider types.

**Discussion and Implications:**

This study provides a better understanding of the difference in the determinants of switching and leaving and simultaneously increases our understanding of the differences between women and men and across provider types.

**Translational Significance:** Although most studies have not separated turnover of direct care workers (DCWs) into those who switch to another organization (switchers) and those who leave the industry (leavers), leavers aggravate the shortage not only in the facilities they quit but also in the entire DCW labor market. Our findings indicate that the impacts of an increase in wages and a higher training score were larger for intent to switch than intent to leave, and that, compared with wages, the impact of training was greater. The findings also have important implications for the design of policies to improve working conditions of DCWs.

## Background and Objectives

Direct care workers (DCWs) help people primarily by assisting with activities of daily living (e.g., eating, bathing, and dressing) and instrumental activities (e.g., shopping and washing). Their role is essential in providing hands-on care for the most frail and vulnerable members of our society. However, a shortage of DCWs is common in most developed countries (Hussein & Manthorpe, 2005; Stone, 2019). The growing demand for workers in aged care, combined with the shrinking supply of younger workers entering the field, portends a future workforce crisis.

Japan, which is the fastest-aging country, has already suffered from a severe shortage of DCWs. DCWs in Japan are equivalent to nursing assistants, home health aides, or personal care aides in the United States. The ratio of job openings per applicant for DCW jobs, which indicates the balance of demand and supply in the labor market, stood at 4.33 in 2019 (Ministry of Health, Labor and Welfare [MHLW], 2018). A national survey showed that 69.7% of care facilities and adult day services and 81.2% of home-based care agencies reported a shortage of DCWs (Care Worker Foundation, 2019). The central government predicted a shortage of 370,000 DCWs nationwide by 2025 (MHLW, 2015).

Organisation for Economic Co-operation and Development (OECD, 2020) demonstrates that DCWs experience low pay and a lack of training. This study investigates the impact of wages and training on turnover intention by sex and provider type (residential care, group home, adult day services, and home-based care). Furthermore, we divide those engaged in voluntary turnover into “switchers,” who continue to work as DCWs but for a different organization, and “leavers,” who work as non-DCWs or who leave the workforce entirely.

### Related Literature

This study makes several contributions to the literature, including the division of voluntary turnover into switching and leaving. Most of the research does not make this distinction, a rare exception being the study by Rosen et al. (2011), who found that the determinants of switchers and leavers were different. Switchers left for new opportunities, whereas leavers left for health reasons. However, Rosen et al. (2011) combined switchers and leavers into one group in a multivariate analysis due to the small sample size. Dill et al. (2013) and Stearns and D’Arcy (2008) examined the determinants of intent to stay in the occupation of DCW. Intent to stay in the occupation seems to relate to intent to leave. However, neither study identified the determinants of intent to switch.

Second, DCWs receive low wages, which contributes to the lower appeal of such jobs (OECD, 2020). Information about the impact of wage increases on turnover is important for policymakers because they have some room for wage increases through subsidies, such as the Medicaid wage pass-through policy, but at the same time they face severe budget constraints. If the impact is small, policymakers should resort to nonpecuniary rewards. However, since many studies examining the effect of wages on turnover have used worker perception rather than actual measures for wages (Brannon et al., 2007; Castle et al., 2007a; Dill et al., 2013), the usefulness of these studies may be somewhat limited. Furthermore, with the exceptions of Howes (2005) and Morris (2009), most of the studies on actual wages reported only the statistical significance of the wage effect or did not compare the effect size of the wage increase with those of other job characteristics (Baughman & Smith, 2012; Rosen et al., 2011; Stearns & D’Arcy, 2008). We add to the literature by predicting the impact of a wage increase on turnover intention with actual hourly wages and compare its effect size with those of other job characteristics, such as training and flexible shifts.

In addition to improving care quality (Institute of Medicine, 2001), training may contribute to the retention of DCWs (Brannon et al., 2007; Castle, et al., 2007a; Coogle et al., 2007). However, most worker-level studies rely on worker perception such as training satisfaction rather than actual measures for training and do not indicate how effective providing training would be for improving retention (Brannon et al., 2007; Castle et al., 2007a; Wiener et al., 2009). Hence, this study used an objective measure for training.

DCWs are overwhelmingly women. Men account for only approximately 20% (Care Worker Foundation, 2019). Due to the small sample size of men, previous studies have not dealt with men separately from women. However, the associations between job attributes and turnover may differ by gender. For example, men may be more sensitive to wages since they are more often breadwinners than women are. Previous studies found lower labor supply elasticity for women compared with men (Barth & Dale-Olsen, 2009; Hirsch et al., 2010), although their study groups consist of a broader set of workers. Furthermore, given the predicted future severe shortage of DCWs, the lack of research on men cannot be overlooked. The large sample size of our study enabled us to analyze male DCWs.

Despite the movement toward more home- and community-based care, most of the research on turnover among DCWs has been conducted in nursing homes and assisted living facilities. Very little work has been done on adult day services or group homes for people with dementia. Adult day services offer a variety of services for infirm older people during daytime hours. They give family caregivers respite from caregiving responsibilities, enabling them to remain in the workplace and continue to give care at home (Fields et al., 2014). Brannon et al. (2007) provide a rare study on the turnover of adult day service workers, but the small sample size hindered statistical analysis. To the best of our knowledge, Suzumura et al. (2013) is the only study that has investigated the determinants of turnover among DCWs in group homes for older people with dementia. However, their study group is restricted to DCWs in group homes located in a large city in Japan. We add to the literature by examining adult day services and group homes with a nationwide survey. Furthermore, we contribute to the literature on home-based care, too. Except for Stone et al. (2017), there is no study on home-based care with a nationwide survey.

## Research Design and Methods

### Conceptual Model

**Figure 1.**
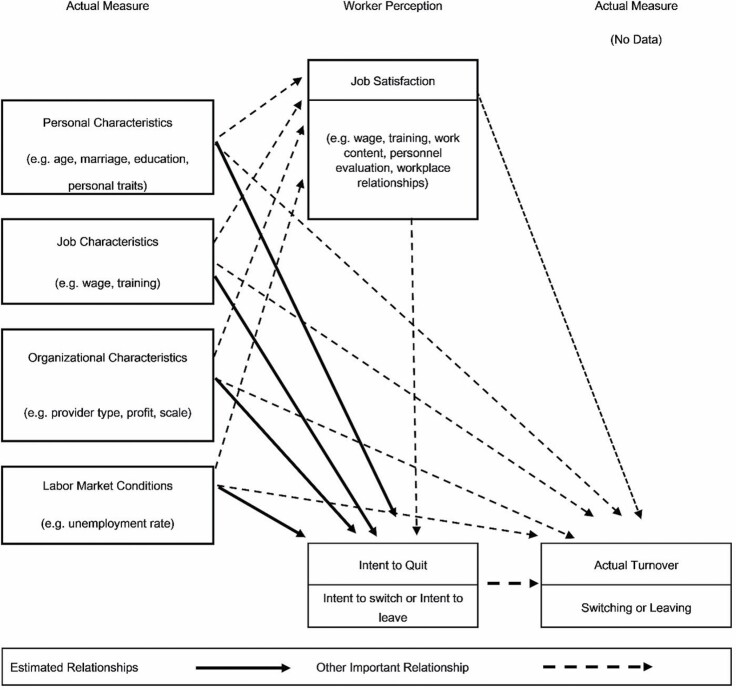
Conceptual model for intent to quit and actual turnover among direct care workers.

Figure 1 summarizes the conceptual model for this study, which is closely aligned with the model used by Castle et al. (2007a). This model hypothesizes that job satisfaction is an important determinant of intent to quit and actual turnover. This has been supported by many studies (Clark, 2001; Lévy-Garboua et al., 2007; Shields & Ward, 2001). However, although our data contain information about job satisfaction, we do not include the data in our analyses. Our interest is in the total effects of wages and training on turnover intention and not the direct effects. Moreover, like Stearns and D’Arcy (2008), we viewed job satisfaction as being simultaneously determined with turnover intention. As described below, we use job satisfaction only for an ancillary analysis.

### Source of Data

The data used here are from the Fact-Finding Survey on Long-term Care Work (FSLCW), which is a nationwide repeated cross-sectional survey in Japan of employees in workplaces that provide long-term care insurance services. The data were provided by the Social Science Japan Data Archive, Center for Social Research and Data Archives, Institute of Social Science, University of Tokyo. The details of the FSLCW sampling method are described in Supplementary Appendix A. The survey asked workers to report (a) demographic information; (b) on-the-job factors, including pay, training, and flexible shifts; (c) attitudinal factors, including job satisfaction, work worries, and reasons for choosing their current workplace; (d) turnover intentions; and (e) characteristics of establishment, including entity, provider type, and number of employees. We used the 2017 and 2018 rounds, which have information about a variety of training programs in the workplace to increase care ability.

A feature of Japanese DCW employment is the extensive use of “nonregular” employees, who are excluded from this analysis. The reasons for the exclusion are as follows. First, in Japan, there are considerable differences between regular and nonregular employees. Regular employees play a central role in their workplaces (Gordon, 2017; Houseman & Osawa, 2003; Kim, 2010). Although regular employees (sei-shain) work with full-time permanent employment contracts, nonregular employees work part-time or have fixed-term employment contracts. The tasks of nonregular employees are basically supplementary to those of regular employees in the same workplace. Across the board, nonregular work offers much lower wages than regular employment. Additionally, nonregular employees have much less job security than regular employees, limited access to training, and weak career prospects. Notwithstanding, about 80% of them accepted nonregular jobs for positive reasons, such as ease of work and low responsibility (MHLW, 2004), suggesting that there are differences in the reason for working between regular and nonregular employees. To ensure the homogeneity of our study group, we excluded 6% of regular employees and included 10% of nonregular employees. The details of this modification are described in Supplementary Appendix B. Second, the sample sizes of nonregular employees by sex and provider type were small. Eventually, we excluded 39% of the observations, and the number of remaining observations that have no missing data for the variables of interest was 7,311.

### Provider Type

We performed the analyses by provider type (residential care, group home, adult day services, and home-based care). The residential care covered by the Japanese public long-term care insurance program comprises three types of facility: nursing homes, health facilities for older people, and designated long-term care beds in hospitals. Health facilities for older people are intermediary ones between hospitals, homes, and nursing homes. Neither the characteristics of the residents nor the actual care they receive differ greatly among these three facilities (Campbell & Ikegami, 2000). Hence, we treated them as one group: “residential care.”

### Variables and Measures

Many studies have examined the determinants of DCW turnover to deal with DCW shortages. Most of them, especially written in English, have been done in the United States. Hence, most of the studies reviewed in this section are for the United States.

#### Dependent variables.

Turnover intention comprises “stay,” “switch,” “leave,” and “not sure.” We defined these by combining the choices for the answer to a question about turnover intention as follows: “Stay” is (a) “I want to remain in my current workplace.” “Switch” is (b) “I want to move to another care workplace,” (c) “I want to move to a welfare workplace other than care,” or (d) “I want to move to a medical facility.” “Leave” is (e) “I want to move to a workplace not related to medicine or care” or (6) “I want to stop working entirely.” “Not sure” is “not sure.” Given potential concerns about (c) and (d) being categorized as “switchers,” we investigated results categorizing them as “leavers.” The percentages of selecting (c) and (d) were only around 2% and 1%, respectively, and the results of the regression analyses for robustness where (c) or (d) is included in “leave” were almost the same (results not shown). The rates of uncombined turnover intentions are shown in Supplementary Table S3.

#### Predictor and control variables.

The variables of key interest here include the wage rate (logged) and training score. The wage rate was constructed using monthly wages and hourly working hours. We treated outliers of wages and hours as missing values.

The survey asked the respondents six questions to assess whether their workplaces had training or mechanisms to increase care ability (off-the-job training, on-the-job training, case studies, task allocations that match workers’ care ability, a care ability evaluation system, payment according to care ability or qualifications or both). Each question was answered with yes or no. We constructed a training score as a count of the number of the times the DCWs answered yes to these questions. The score indicates the extensiveness of training. We expected that extensive training programs would reduce turnover intention through an increase in job satisfaction. To confirm the validity of the score, we regressed training satisfaction score on the training score and the other independent variables described in this section. The results showed that a high training score was significantly associated with high training satisfaction (Supplementary Table S4b).

Because research indicates that schedule flexibility is an attractive job-related factor (Howes, 2008; Morris, 2009), we included a dichotomous variable for flexibility. The survey asked the respondents whether they had opportunities to express their preferences to their supervisors about scheduling shifts (1 = yes, 0 = no).

Wages, training, and flexibility are more objective than worker perceptions, such as job satisfaction. Economists have been treating perceptions as endogenous variables (Chen et al., 2013; Dionne et al., 2007; Viscusi, 1991). For example, unobservable personal traits, such as diligence, might correlate with training satisfaction and turnover. Thus, our objective measure may be less susceptible to endogeneity bias. However, some unobservable personal traits may be correlated with our objective measures. For example, people whose intrinsic motivation is high might tend to choose workplaces that provide extensive training programs. Furthermore, people who make much of their work–life balance might tend to choose workplaces that afford them a flexible working style. If so, the impact of wages, training, and flexibility on turnover intention could be overestimated. Hence, to control for potential unobservable confounding factors, we included three binary variables (0/1), each indicating a reason for choosing the current workplace: the wages were good, the training was substantial, and the workdays and hours were in line with the respondent’s preferences.

Previous research has found that supportive supervision (positive, respectful, and helpful interaction with one’s supervisor) decreases turnover intention (Bethell et al., 2018; Bishop et al., 2008; Stearns & D’Arcy, 2008). Unfortunately, our data contain no information about supportive supervision. Therefore, we created a proxy measure for supportive supervision through the responses to two questions, one concerning whether the respondents had opportunities to ask their supervisors for advice regarding the content of their work, work style, and career advancement, the other concerning whether their workplace had a consultation support system. Both were measured using dichotomous variables (1 = yes, 0 = no). A proxy measure was created by adding these two variables. We expected that this measure would correlate with supportive supervision and designated the measure “support.”

The personal variables were age (logged), marital status, education, and primary earners in the household. The role-related variables were tenure in the current workplace (logged), nonregular employee, duty position, and qualification as a so-called certified care worker. The certified care worker is a national top-level qualification and is not a licensed occupation, but holders are registered and certified as having the right to a title (Gospel, 2015). Training to be a certified care worker is displayed in Supplementary Figure S1.

Facility characteristics are controlled for by for-profit ownership and facility size. Labor market conditions are controlled for by including city size and unemployment rates by prefecture and a year dummy. Unemployment rates come from the Statistics Bureau, Ministry of Internal Affairs and Communications and are linked to workers according to their workplace address.

### Analyses

The dependent variable is turnover intention, which consists of four categories (“stay,” “switch,” “leave,” and “not sure”). Hence, we used a multinomial logit model, when the dependent variable is nominal and for which there are more than two categories (Greene, 2018).

## Results

### Descriptive Statistics

Table 1 presents the descriptive statistics of the sample of DCWs whose payments were monthly (*N* = 6,716). We omitted the results of male group home workers (*n* = 303) and male home-based care workers (*n* = 292) because their sample sizes were rather small. The descriptive statistics of these settings are displayed in Supplementary Table S5. The descriptive statistics for the full sample are displayed in Supplementary Table S6. The proportion of males was highest in residential care (41%) and lowest in home-based care (20%). With regard to turnover intention, the proportion of the study group who expected to “stay” was the lowest among female residential care workers (46%), highest in female home-based care workers (62%), and 52%–56% for others. The proportion of “switchers” were about 1.6 times higher than “leavers” in all cases. About one in four observations were “not sure” in all cases. The Cronbach’s coefficient alphas of training were .70–.74. Compared with women, men were younger, more highly educated, more likely to be breadwinners, and in higher positions. The distributions of training scores were similar across service types (Supplementary Table S4c). Interestingly, men had more opportunities for training than women. Approximately 50%–60% of respondents across provider-specific groups had opportunities to express their preferences to their supervisors about scheduling shifts.

**Table 1.** Descriptive Statistics

Variable	Residential care		Group home	Adult day service		HBC
	Women	Men	Women	Women	Men	Women
*N*	1,376	949	819	1,753	635	1,184
Gender ratio	59%	41%	73%	73%	27%	80%
Dependent variable						
Turnover intention						
Stay	46%	53%	52%	56%	56%	62%
Switch	17%	13%	14%	12%	13%	9%
Leave	10%	8%	10%	7%	8%	6%
Not sure	27%	27%	24%	26%	24%	23%
Independent variables						
Job characteristics						
Hourly wage (yen), mean	1,164	1,249	1,108	1,036	1,131	1,109
Training (range: 0–6), mean	2.8	3.2	2.7	2.6	3.0	3.0
Reliability coefficients for training, α	0.716	0.709	0.707	0.706	0.739	0.723
Flexible shift	53%	58%	61%	53%	57%	56%
Support (range: 0–2), mean	0.63	0.73	0.64	0.66	0.80	0.78
Role-related characteristics						
Tenure, mean	8.3	7.6	7.1	6.2	5.9	6.8
Duty position						
Manager	3%	5%	10%	5%	12%	15%
Assistant manager	44%	51%	32%	22%	25%	15%
Other	53%	43%	58%	73%	64%	71%
Certified Care Worker	81%	80%	74%	68%	60%	65%
Nonregular	6%	2%	6%	9%	4%	11%
Personal characteristics						
Age (mean)	41	36	46	43	38	47
Marriage	41%	58%	43%	56%	49%	53%
Breadwinner	38%	67%	45%	34%	61%	41%
Education						
High school	66%	53%	71%	65%	51%	72%
Junior college	24%	19%	20%	26%	18%	21%
College	10%	28%	9%	9%	32%	7%
Reasons to choose the current workplace						
Wage	10%	11%	6%	8%	7%	11%
Training	2%	2%	2%	2%	3%	2%
Working hour or day	9%	6%	9%	30%	18%	20%
Facility characteristics						
Nonprofit^a^	—	—	45%	51%	54%	38%
Scale^b^						
Small	49%	43%	—	31%	25%	35%
Middle	36%	39%	—	33%	32%	31%
Large	15%	18%	—	36%	43%	34%
Turnover opportunity						
Unemployment rate, mean	2.4	2.5	2.5	2.4	2.5	2.6
Population size						
Large city^c^	13%	18%	17%	16%	22%	25%
Small and medium city	67%	65%	65%	68%	65%	59%
Village	20%	18%	19%	16%	14%	17%
Year 2018	51%	47%	43%	48%	51%	45%

*Notes:* HBC = home-based care.

^a^In Japan, only nonprofit entities are allowed to supply residential care.

^b^Number of employees per establishment. Institutional care: middle (20–49), large (50–). Adult day service and home-based care: middle (10–19), large (20–). Because the numbers of group home employees were small, the scale was omitted.

^c^Tokyo 23 wards and ordinance-designated city.

### Regression Results

Table 2 reports the results from the multinomial regression model examining turnover intention. To save space, the results of “not sure” are omitted. The results of the full model including the coefficients for the “not sure” are displayed in Supplementary Tables S7a–d. Overall, the sign of the coefficients of job characteristics was in the expected negative direction. However, the size and significance level of the coefficients varied between intent to switch and leave, between men and women, and across provider types. The results of male group home and home-based care workers are displayed in Supplementary Tables S7b and d, respectively. Table 3 displays the projected effects on the probability that a DCW will be in a group with the intention to switch or leave in response to changes in each independent variable.

**Table 2.** Multinomial Logistic Regression Results for Turnover Intention

Variable	Residential care				Group home		Adult day service				Home-based care	
	Women		Men		Women		Women		Men		Women	
	Switch	Leave	Switch	Leave	Switch	Leave	Switch	Leave	Switch	Leave	Switch	Leave
Job characteristics												
Hourly wage (logged)	−1.18**	−1.13*	−1.36**	−1.89**	−0.81	−1.02	−2.35**	−0.88	−2.80**	−3.48**	−0.77	−2.46**
Training	−0.12*	−0.24**	−0.11	−0.15	−0.34**	−0.23**	−0.14*	0.07	−0.15	−0.25*	−0.17*	−0.06
Flexible shift	−0.03	−0.33	−0.48*	−0.57*	−0.15	−0.12	−0.40+	−0.28	−0.39	0.06	−0.70*	−1.09**
Support	−0.36**	−0.24	−0.39*	−0.17	−0.27^+^	−0.28	−0.14	−0.74**	−0.39^+^	−0.48*	−0.30^+^	−0.43^+^
Role-related characteristics												
Tenure (logged)	−0.02	0.35*	0.09	−0.04	−0.08	0.29^+^	−0.01	0.10	−0.03	0.52*	0.07	0.18
Manager	0.43	0.10	−0.11	−1.20	−0.12	0.03	−0.96	0.51	0.15	1.23*	−0.13	−0.11
Assistant manager	0.13	−0.53*	−0.10	−0.04	0.12	0.14	0.38^+^	−0.04	0.35	0.28	−0.12	0.34
Certified care worker	0.20	−0.08	−0.05	−0.51	0.36	0.25	0.32	0.42	0.09	−0.37	0.24	0.11
Nonregular	−0.51	−0.26	−1.30	−0.97	0.37	0.78	−0.30	−0.19	0.34	0.63	−0.26	−0.51
Personal characteristics												
Age (logged)	−1.39**	−1.14*	−0.67	0.06	−1.99**	−2.12**	−1.47**	−0.52	−1.05**	−0.73	−0.87*	0.17
Marriage	−0.40*	−0.62*	−0.24^+^	−0.61*	0.30	0.14	0.05	−0.24	0.49	−0.05	−0.87**	−0.73^+^
Breadwinner	0.33^+^	0.03	0.29	0.52	0.87**	0.51^+^	0.65**	0.26	−0.49	0.27	−0.31	−0.24
Junior college	0.13	−0.25	0.07	0.14	0.24	0.25	−0.10	−0.05	−0.21	−0.25	0.06	−0.21
College	0.82**	0.57**	0.12	−1.38**	0.33	0.24	0.45*	0.33	−0.09	0.20	0.18	−0.57
Reasons to choose the current workplace												
Wage	−0.27	0.24	−0.30	−0.23	0.13	−1.51	0.00	−0.53	0.30	0.98	0.38	0.07
Training	−1.20*	−0.61	0.54	−0.09	−0.04	0.79	−0.27	−0.47	−0.57	−12.8**	0.10	−12.7**
Working hour or day	0.02	−0.04	−0.86^+^	−1.75^+^	−1.30*	−1.05*	−0.26	−0.16	−0.80^+^	−0.47	−0.45	−0.50
Facility characteristics												
Nonprofit	—	—	—	—	0.15	−0.09	0.00	0.35	0.22	−0.46	−0.07	−0.21
Scale: middle^a^	0.22	−0.36	−0.19	0.27	—	—	0.16	−0.13	0.75^+^	−0.21	0.29	0.52^+^
Scale: large^a^	0.07	0.11	0.00	−0.03	—	—	0.17	−0.10	0.72^+^	−0.33	0.24	0.03
Turnover opportunity												
Unemployment rate	0.02	−0.03	0.16	0.02	0.03	0.25	0.04	−0.15	0.41	0.13	−0.06	−0.49^+^
Large city^b^	0.37	−0.30	−0.24	0.28	0.44	−0.20	−0.25	−0.00	1.67**	0.55	−0.22	0.16
Small and medium city^b^	0.17	−0.35	−0.22	−0.03	0.46*	0.15	−0.09	0.33	1.18*	0.11	−0.13	0.06
Year 2018	−0.13	−0.14	0.38	−0.13	0.10	0.10	0.08	−0.02	0.56^+^	0.64^+^	0.61^+^	−0.12
Pseudo-*R*^2^	0.067		0.067		0.090		0.065		0.110		0.089	
*N*	1,376		949		819		1,753		635		1,184	

*Notes:* The reference category of the dependent variable is “stay.” In account for the possible correlation of variables within prefecture, we used the Huber–White sandwich estimator clustered by prefecture.

^a^Number of employees per establishment. Residential care: middle (20–49), large (50–). Adult day service and home-based care: middle (10–19), large (20–).

^b^Tokyo 23 wards and ordinance-designated city.

^+^ *p* ≤ 0.1.

*p ≤ .05.

**p ≤ .01.

**Table 3.** Estimated % Reduction in Turnover Intentions with Simulated Hourly Wage and Training Score Increase

Variable	Residential care				Group home		Adult day service				Home-based care	
	Women		Men		Women		Women		Men		Women	
	Switch	Leave (total)	Switch	Leave (total)	Switch	Leave (total)	Switch	Leave (total)	Switch	Leave (total)	Switch	Leave (total)
10% increase	−1.1	−0.6 (−1.7)	−0.9	−0.7 (−1.6)	−*0.6*	−*0.7* (−*1.3*)	−1.6	−*0.2* (−1.8)	−1.7	−0.9 (−2.6)	−*0.3*	−0.6 (−0.9)
20% increase	−2.1	−1.1 (−3.2)	−1.7	−1.2 (−2.9)	− *1.2*	−*1.2* (−*2*.*4*)	−3.0	−*0.4* (−3.4)	−3.1	−1.6 (−4.7)	−*0.6*	−1.1 (−1.7)
30% increase	−3.0	−1.5 (−4.5)	−2.4	−1.7 (−4.1)	−*1.7*	−*1.7* (−*3.4*)	−4.0	−*0.7* (−4.7)	−4.2	−2.1 (−6.3)	−*0.9*	−1.4 (−2.3)
Training score change												
From 0 to 1	−1.0	−2.3 (−3.3)	−*0.6*	−*0.6* (−*1.2*)	−4.0	−1.1 (−5.1)	−1.0	*0.7* (−*0.3*)	−*0.4*	−0.8 (−1.2)	−1.6	−*0.1* (−1.7)
From 1 to 2	−1.1	−2.0 (−3.1)	−*0.6*	−*0.6* (−*1.2*)	−3.5	−1.1 (−4.6)	−1.0	*0.7* (−*0.3*)	−*0.5*	−0.8 (−1.3)	−1.4C	−*0.1* (−1.5)
From 2 to 3	−1.1	−1.7 (−2.8)	−*0.7*	−*0.6* (−*1.3*)	−3.0	−1.2 (−4.2)	−1.0	*0.8* (−*0.2*)	−*0.6*	−0.7 (−1.3)	−1.2	−*0.1* (−1.3)
From 3 to 4	−1.1	−1.5 (−2.6)	−*0.7*	−*0.5* (−*1.2*)	−2.5	−1.1 (−3.6)	−1.0	*0.8* (−*0.2*)	−*0.7*	−0.7 (−1.4)	−1.1	−*0.1* (−1.2)
From 4 to 5	−1.1	−1.2 (−2.3)	−*0.7*	−*0.5* (−*1.2*)	−2.1	−1.1 (−3.2)	−0.9	*0.9* (−*0.0*)	−*0.7*	−0.6 (−1.3)	−1.0	−*0.1* (−1.1)
From 5 to 6	−1.1	−1.0 (−2.1)	−*0.7*	−*0.5* (−*1.2*)	−1.6	−1.0 (−2.6)	−0.9	*0.9* (−*0.0*)	−*0.8*	−0.5 (−1.3)	−0.8	−*0.1* (−0.9)
From 2 to 6	−4.5	−5.5 (−10.0)	−*2.7*	−*2.1* (−*4.8*)	−9.2	−4.4 (−13.6)	−3.8	*3.5* (−*0.3*)	−*2.8*	−2.6 (−5.4)	−4.1	−*0.4* (−4.5)
From 0 to 6	−6.6	−9.8 (−16.4)	−*3.9*	−*3.3* (−*7.2*)	−16.8	−6.6 (−23.4)	−5.8	*4.9* (−*0.9*)	−*3.7*	−4.2 (−7.9)	−7.0	−*0.6* (−7.6)
From 0 to 1	−*2.1*	−*1.5* (−*3.6*)	−4.3	−2.6 (−6.9)	−*0.4*	−*0.0* (−*0.4*)	−3.8	−*1.5 (*−5.3)	−*3.7*	*0.4* (−3.4)	−3.6	−3.0 (−6.6)
From 0 to 1	−3.4	−*0.7* (−4.1)	−3.6	−*0.3* (−3.9)	−1.0	−*0.8* (−1.8)	−*0.1*	−4.6 (−4.7)	−3.6	−2.0 (−5.6)	−1.6	−1.2 (−2.8)
From 1 to 2	−3.1	−*0.8* (−3.9)	−2.8	−*0.4* (−3.2)	−1.4	−*1.1* (−2.5)	−*0.4*	−2.6 (−3.0)	−2.8	−1.4 (−4.2)	−1.5	−0.9 (−2.4)
From 0 to 2	−6.4	−*1.5* (−7.9)	−6.4	−*0.7* (−7.1)	−2.4	−*1.9* (−4.3)	−*0.5*	−7.2 (−7.7)	−6.5	−3.4 (−9.9)	−3.1	−2.2 (−5.3)
Base case intention	17	10 (27)	13	8 (21)	14	10 (24)	12	7 (19)	13	8 (21)	9	6 (15)
Mean wage	1,164 yen		1,249 yen		1,108 yen		1,036 yen		1,131 yen		1,109 yen	

*Notes*: The simulated impact of wage increases is measured as the percent change in predicted turnover intention compared with workers earning the average hourly wage. All estimates are based on the parameters displayed in Table 2, holding all other variables at their means. Base case intention is predicted turnover intention based on all the mean independent variables. Numbers in parentheses are the sum of switch and leave. Italic numbers are based on insignificant results.

Table 3 indicates that overall, the increase in hourly wage reduced the likelihood of intent to switch and intent to leave but that the size of the effect on intent to switch was larger than it was for intent to leave in all but two comparisons. This was most clearly evident for female adult day service workers, in which the ratio of the predicted reduction of intent to switch to the predicted reduction of intent to switch and leave was about 0.9 (e.g., 3.0/[3.0 + 0.4] in the case of a 20% wage increase). Examination of the four provider-specific subsamples showed that the wage impact was not equally distributed. DCWs in adult day services showed more of a connection between wage increase and turnover intention than did the other provider types.

A higher training score also reduced both intent to switch and intent to leave. The impact was greater for the intent to switch. Although the simulated impact of a 1-point increase in training score on turnover intention gradually decreased as the score increased, even the lowest reduction in intent to switch with a 1-point increase (5–6) on the score was the same as or larger than the predicted reductions with a 10% wage increase in all but adult day service and male residential care workers. Because Supplementary Table S4c showed that the training scores of about half of the DCWs were 2 or below, Table 3 shows the predicted reduction in intent to switch and intent to leave with a 4-point increase in the score (2–6). The sizes of the reductions on intent to switch were larger than the reduction with a 30% wage increase all but adult day service workers. With regard to intent to switch, the strongest impact in the group home workers saw the lowest reduction in intent to switch with a 1-point increase in the score (5–6), at 1.6% points, whereas the maximum attainable reduction, with a 6-point increase (0–6), was 16.8% points, which was more than double that of the other provider types. The training score was statistically associated with intent to leave for female residential care, female group home, and male adult day service workers. For those groups, the size of the reductions in intent to leave, with a 4-point increase in the score (2–6), was larger than the reduction with a 30% wage increase. For the other groups, the reduction in intent to leave with flexible shifts was more than three times larger than the predicted reductions with a 10% wage increase. The strongest effect was in the home-based care workers, with 3.6%-points and 3.0%-points reductions in intent to switch and intent to leave, respectively. Overall, support reduced turnover intention. There were some differences between women and men. In residential care, the training score was not statistically associated with intent to leave for men, whereas flexible shift was not statistically associated with intent to switch for women. The complete opposite propensity was found in adult day services.

### Sensitivity Analyses

Leavers are composed of individuals who want to switch to a nonhealth care setting and want to leave the labor force altogether. However, combining these two groups could be inadequate because the determinants of these turnover intentions could be different. Furthermore, women who have children may be more likely to want to leave the labor force. However, our data contain no information about the children of DCWs. Hence, we first conducted regressions separating these two groups (Supplementary Table S8). As for wage and training, we compared the differences in the coefficients between these two groups by gender and provider type with the Wald test. In all comparisons, no differences were statistically significant at any conventional level all but group home workers. Furthermore, we added another independent variable to the above regressions. The variable takes the value of 1 if workers are married women in their thirties, and 0 otherwise. We expected that this binary variable would capture the influence of children in the household on the women labor force participation to some extent because the labor participation rate of Japanese women decreases in their 30s due to childrearing. Specifically, the labor participation rate of Japanese females decreased from 82% in their late 20s to 75% in their 30s and increased again to 79% in their early 40s in 2019 (MHLW, 2020). However, the added variable hardly changed the coefficients of the two key variables (results not shown). Hence, we concluded that combining the two groups is not inappropriate, and the lack of information about children has little effect on our main results.

## Discussion and Implications

Both switchers and leavers increase the replacement costs of recruiting new employees (Leon et al., 2001) and interfere with quality of care (Castle et al., 2007b; LaPrante et al., 2004). However, because leavers decrease the number of active DCWs in the labor market, they aggravate the shortage not only in the facilities they quit but also in the entire labor market for DCWs. By contrast, although switchers cause problems for the care facilities they quit, their impact on the labor market for DCWs may not be as great. Furthermore, hiring a DCW without prior experience is likely to be more costly in terms of the initial training required than hiring an experienced DCW. Hence, the replacement cost caused by leavers may be higher than that caused by switchers. Therefore, the prevention of switching and leaving has different implications. Most previous studies have not distinguished between switchers and leavers, however. This is the first study to examine the determinants of the likelihood of intending to switch and intending to leave using a multiple regression analysis. This is also the first study to deal with men separately from women in research on DCW turnover. These analyses were possible because of the large size of our data.

The descriptive analysis showed that the proportions of those who intended to switch were about 1.7 times higher than those who intended to leave in all provider types. This finding seems to reflect the large degree of mobility of DCWs within the field of long-term care (Baughman & Smith, 2012).

The key finding from this analysis is that the sizes of the impacts of job characteristics on intent to switch and intent to leave are different. Overall, the projected effect on the likelihood of intent to switch in response to changes in each job characteristic independent variable was larger than it was for intent to leave. In addition, the predicted reduction in turnover intention with nonpecuniary job characteristics, training, and flexible shift was larger than the reduction with wage increases. Furthermore, both training and flexible shifts were not prevalent, which suggests that there is much room for improvement with regard to those job conditions. Considering the severe budget constraints for wage increases, training and flexible shifts would seem to be promising and cost-effective measures for the retention of DCWs.

Japanese policy recognizes the importance of DCW wages and training for retention. To raise the wages of DCWs, the Japanese government introduced a subsidy to employers to improve the wages for care workers in 2009 (Ohkubo, 2018). Although the subsidy was converted to public long-term care insurance service fee additions in 2012, the fee served the same function as the subsidy (Supplementary Figure S2). Based on several conditions, fee additions vary from 120,000 yen ($1,100) to 370,000 yen ($3,300) for wage increases for DCWs. All fee additions must be used for wage increase in DCWs. The DCWs qualified for a wage increase are those who work for establishments that provide long-term care insurance services. To obtain over 27,000-yen fee additions, employers must offer DCW training to improve their ability. If not, the fee additions are at best 150,000 yen; 85.6% of the establishments received over 27,000 yen in 2020. However, this does not stipulate anything about the type and amount of training. If the amount of the subsidy is set according to the clearly defined extensiveness of training, employers could improve their training programs. Both Japan and the United States have been suffering from a severe shortage of DCWs, and the above subsidy in Japan and the Medicaid wage pass-through policy in the United States are similar in that both are for increasing the wages. Hence, the Japanese system might have important policy implications for the United States.

Due to their small sample sizes of men, previous studies have not dealt with men separately from women. However, given the predicted future severe shortage of DCWs, the lack of research on men cannot be overlooked. The results show that the association between training and turnover intention and the association between flexible shifts and turnover intention were clearly different between women and men. These findings suggest that management practices useful for the prevention of female turnover may not be relevant to males.

Despite the movement toward more home- and community-based care, very little work has been done on turnover among adult day services and group home workers. The results indicate that the reduction in intent to switch with changes in training score in group homes was almost twice that of the other service types. Adult day service workers were more responsive to wage increases.

Turnover intention is a predecessor to actual turnover. Therefore, if the social and economic environments surrounding DCWs dramatically changed, the discrepancy between turnover intention and actual turnover would widen. For example, the shortage of DCWs in Japan, seriously worsening in the late 1990s, was sharply eased due to the Great Recession (Hanaoka, 2015). The survey used in this study was conducted before the COVID-19 pandemic. Additionally, now amidst the COVID-19 pandemic, different forces may lead to workers making different choices about work. For example, deteriorating working conditions, such as the risk of infection (Almeida et al., 2020; Bandini et al., 2021), could lead to the outflow from the labor market of DCWs. On the other hand, the decrease in labor demand in other fields could narrow the opportunities for DCWs to move to other fields. As of October 2021, in Japan, the shortage of DCWs is as severe as before the pandemic. This might suggest that the opposite forces counterbalance. In short, we do not find any clear evidence that the discrepancy between turnover intention and actual turnover has widened.

### Limitations and Future Research

One limitation of this study is that the cross-sectional nature of the data set does not allow for an assessment of actual turnover. Although turnover intentions and actual turnover are highly correlated (Alexander et al., 1998; Hendrix et al., 1999; Rosen et al., 2011), intent to quit alone accounts for only a portion of actual turnover (Mor Barak et al., 2001). Another limitation relates to the observational nature of this study. Although we tried to prevent endogeneity biases by including reasons to choose the current workplace, and by using an objective scale rather than worker perceptions, potential biases could remain. In that case, the estimates cannot be interpreted as pure effects of job characteristics.

Our training score does not consider the frequency and intensity of training. Hence, there is room for improvement in the training score used here. We excluded most of the nonregular workers, which consist of 40% of our observations. This rate is never negligibly small. Furthermore, considering the large differences between regular and nonregular workers in Japan, it would be very interesting to compare the determinants of turnover intention between them. Hence, research on the turnover intention of nonregular workers should be done in the future.

## Supplementary Material

igac035_suppl_Supplementary_Material
