# Supplementary material for: The Effects of Wages and Training on Intent to Switch or Leave Among Direct Care Workers
Source: Innov Aging. 2022 May 20;6(4):igac035. doi: 10.1093/geroni/igac035 (PMC9273402; doi:10.1093/geroni/igac035)
Supplement: igac035_suppl_Supplementary_Material [file igac035_suppl_supplementary_material.docx]

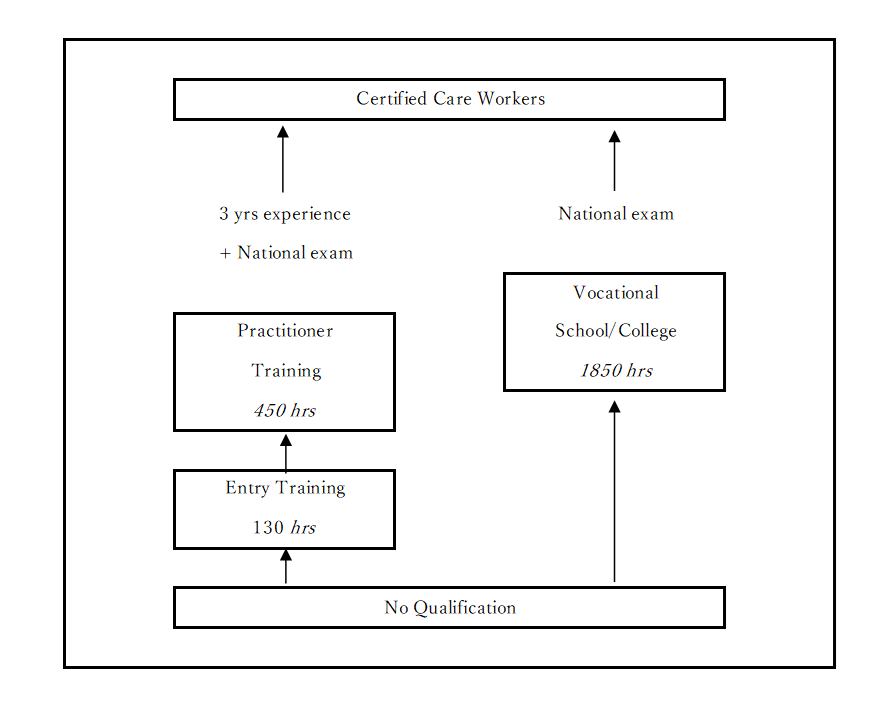


**Figure S1. Training and Examinations Required to Become a Certified Care Worker**

**Figure S2. Outline of the Japanese long-term care insurance system**

^a^ Subsidy to improve the wages for care workers

^b^ Fee additions to improve the wages for care workers

**Appendix A**

Fact-Finding Survey on Long-term Care Work (FSLCW)

The FSLCW is conducted annually by the Care Work Foundation. We used the 2017 and 2018 rounds. In 2017, the sample included 17,638 care establishments randomly selected from all establishments that provided long-term care insurance services. The FSLCW sample used a two-stage design. In the first stage, 8,782 of the 17,638 establishments decided to participate in the survey. In the second stage, the participating establishments chose up to three employees as respondents to answer the questions according to the instructions of the Care Work Foundation. The instructions defined the job and the type of work of the respondents by provider type so that the respondents would be typical employees of the establishments. The respondents were required to return the fill-out questionnaires directly to the Care Work Foundation by postal mail, not through their establishments. A total of 21,250 responses were collected, yielding a combined first- and second-stage response rate of 40%. No sampling weights are used in this survey. The respondents included not only DCWs but also paramedical staff, such as nurses and physical therapists. As described in the material, we used only DCWs whose payments were monthly. The survey method was the same regardless of the survey year. There is a possibility that responses could have been included from the same workers in the two years. However, given the population of DCWs, 1.8 million, that is unlikely.

| **Table S1. Response rates and the number of collected responses** | | |
| --- | --- | --- |
| Survey year | 2017 | 2018 |
| Original care establishments | 17,638 | 18,000 |
| Participating care establishments | 8,782 | 9,102 |
| Employees at the original care establishments (a) | 52,914 | 52,943 |
| Collected responses (b) | 21,250 | 22,183 |
| Response rate (b)/(a) | 40% | 42% |

**Appendix B**

The primary criterion for classifying regular and non-regular workers in Japanese surveys including FSLCW is whether or not they are called non-regular workers at their workplaces. As the criterion is not objective, some regular workers could be virtually non-regular, and vice versa. In our sample, there were apparent differences in wage forms between regular and non-regular workers. While 94 % of regular workers were paid monthly (Table S2a), 85 % of non-regular workers were paid on an hourly basis. We exclude regular employees paid on non-monthly basis since their working hours and duty positions were similar to non-regular employees in adult day service and home-based care (data not shown). On the other hand, we include non-regular workers paid every month since their working hours were almost the same as regular workers (data not shown). It has been highlighted that the number of full-time non-regular employees regarded as performing similar tasks as regular workers has been rapidly increasing in the 2000s in Japan (Kim, 2010). Eventually, our targets are regular and non-regular employees with monthly payments.

| **Table S2a. Breakdown of Pay Form by Regular and Non-regular Workers (%)** | | |
| --- | --- | --- |
|  | Regular workers | Non-regular workers |
| Monthly | 94 | 10 |
| Hourly | 4 | 85 |
| Daily | 1 | 3 |
| Other | 1 | 2 |
| Total | 100 | 100 |

| **Table S2b. Number of Observation by Wage Form, Provider Type and Gender** | | | | | | | | | | | | | |
| --- | --- | --- | --- | --- | --- | --- | --- | --- | --- | --- | --- | --- | --- |
|  | Residential Care | |  | Group Home | |  | Adult Day Service | |  | Home Based Care | |  | Total |
|  | Women | Men |  | Women | Men |  | Women | Men |  | Women | Men |  |  |
| Monthly | 1,485 | 1,043 |  | 848 | 322 |  | 1,880 | 723 |  | 1,317 | 342 |  | 7,960 |
|  | (75%) | (94%) |  | (70%) | (88%) |  | (48%) | (80%) |  | (43%) | (80%) |  | (62%) |
| Hourly | 443 | 51 |  | 332 | 37 |  | 1,930 | 156 |  | 1,577 | 71 |  | 4,597 |
|  | (23%) | (5%) |  | (28%) | (10%) |  | (50%) | (17%) |  | (52%) | (17%) |  | (36%) |
| Daily | 36 | 15 |  | 17 | 7 |  | 71 | 15 |  | 49 | 8 |  | 218 |
|  | (2%) | (1%) |  | (1%) | (2%) |  | (2%) | (2%) |  | (2%) | (2%) |  | (2%) |
| Other | 7 | 6 |  | 11 | 2 |  | 22 | 5 |  | 91 | 9 |  | 153 |
|  | (0%) | (1%) |  | (1%) | (1%) |  | (1%) | (1%) |  | (3%) | (2%) |  | (1%) |
| Total | 1,971 | 1,115 |  | 1,208 | 368 |  | 3,903 | 899 |  | 3,034 | 430 |  | 12,928 |
|  | (100%) | (100%) |  | (100%) | (100%) |  | (100%) | (100%) |  | (100%) | (100%) |  | (100%) |
| *Notes*: Observations used in this study are the ones whose pay form is monthly. | | | | | | | | | | |  |  |  |

| **Table S2c. Breakdown of Employment Type** | | | | | | | | | | | | | |
| --- | --- | --- | --- | --- | --- | --- | --- | --- | --- | --- | --- | --- | --- |
|  | Residential Care | |  | Group Home | |  | Adult Day Service | |  | Home Based Care | |  | Total |
|  | Women | Men |  | Women | Men |  | Women | Men |  | Women | Men |  |  |
| Regular workers | 74% | 94% |  | 73% | 92% |  | 49% | 81% |  | 44% | 81% |  | 62% |
| Non-regular workers | 26% | 6% |  | 27% | 8% |  | 51% | 19% |  | 56% | 19% |  | 38% |

| **Table S3. Breakdown of Turnover Intention (%)** | | | | | | | | | |  |  |
| --- | --- | --- | --- | --- | --- | --- | --- | --- | --- | --- | --- |
|  |  | |  |  |  |  | |  |  |  |  |
|  | Residential Care | |  | GH |  | Adult Day Service | |  | HBC |  | Total |
|  | Women | Men |  | Women |  | Women | Men |  | Women |  |  |
| Current workplace^a^ | 46 | 53 |  | 52 |  | 56 | 56 |  | 62 |  | 56 |
| Other care workplace^b^ | 12 | 9 |  | 11 |  | 8 | 9 |  | 7 |  | 9 |
| Welfare job^c^ | 4 | 3 |  | 2 |  | 2 | 3 |  | 2 |  | 2 |
| Medical workplace^d^ | 1 | 1 |  | 1 |  | 1 | 1 |  | 1 |  | 1 |
| Other job^e^ | 7 | 6 |  | 8 |  | 5 | 6 |  | 4 |  | 5 |
| I want to stop working | 3 | 2 |  | 2 |  | 2 | 2 |  | 2 |  | 2 |
| Not sure | 27 | 27 |  | 24 |  | 26 | 24 |  | 23 |  | 24 |
| *Notes.* GH=Group home. HBC=home-based care.  ^a^“I want to remain in my current workplace.”  ^b^“I want to move to another care workplace.”  ^c^“I want to move to a welfare workplace other than care.”  ^d^“I want to move to a medical facility.”  ^e^“I want to move to a workplace not related to medicine or care.” | | | | | | | | | | | |

| **Table S4a. Breakdown of Training Satisfaction (%)** | | | | | | | | | |
| --- | --- | --- | --- | --- | --- | --- | --- | --- | --- |
|  | Residential Care | | GH | | | Adult Day Service | |  | HBC |
|  | Women | Men |  | Women |  | Women | Men |  | Women |
| Very satisfied (5) | 4 | 3 |  | 5 |  | 4 | 7 |  | 8 |
| Moderately satisfied (4) | 13 | 14 |  | 15 |  | 14 | 17 |  | 15 |
| Neither (3) | 52 | 53 |  | 54 |  | 56 | 51 |  | 58 |
| Moderately unsatisfied (2) | 24 | 21 |  | 19 |  | 20 | 18 |  | 14 |
| Very unsatisfied (1) | 8 | 9 |  | 8 |  | 7 | 7 |  | 6 |
| *Notes.* GH=Group home. HBC=home-based care. | | | | | | | | | |

| **Table S4b. Coefficients for Predictors for Training Satisfaction** | | | | | | | | | | | |
| --- | --- | --- | --- | --- | --- | --- | --- | --- | --- | --- | --- |
|  |  |  | Residential Care | | Group Home | | | Adult Day Service  m | | | HBC |
|  |  |  | Women | Men |  | Women |  | Women | Men |  | Women |
| *Job characteristics* | | |  |  |  |  |  |  |  |  |  |
|  | Hourly wage (logged) | | 0.05 | 0.76+ |  | 0.24 |  | 0.62** | 1.51** |  | 0.74** |
|  | Training | | 0.37** | 0.22** |  | 0.41** |  | 0.35** | 0.38** |  | 0.25** |
|  | Flexible shift | | 0.15 | 0.00 |  | 0.47** |  | 0.11 | 0.22 |  | 0.16 |
|  | Support | | 0.08 | 0.31* |  | 0.17 |  | 0.34** | 0.23* |  | 0.47** |
| *Role-related characteristics* | | |  |  |  |  |  |  |  |  |  |
|  | Tenure | | -0.09 | -0.12 |  | 0.09 |  | -0.02 | -0.12 |  | 0.00 |
|  | Manager  i | | -0.52^+^ | -0.22 |  | -0.14 |  | -0.20 | -0.29 |  | -0.33* |
|  | Assistant manager  i | | -0.19 | -0.32+ |  | -0.22 |  | -0.21 | -0.38 |  | 0.06 |
|  | Certified Care Worker | | -0.15 | 0.01 |  | -0.22 |  | -0.22* | 0.08 |  | -0.12 |
|  | Non-regular | | -0.23 | 0.06 |  | 0.33 |  | 0.01 | 0.53 |  | 0.31+ |
| Personal characteristics | | |  |  |  |  |  |  |  |  |  |
|  | Age (logged) | | -0.23 | -0.67** |  | -0.18 |  | -0.71** | -1.01** |  | -0.72* |
|  | Marriage | | 0.15 | 0.05 |  | 0.13 |  | 0.14 | -0.10 |  | 0.50** |
|  | Breadwinner | | -0.02 | -0.19 |  | -0.02 |  | -0.26* | 0.17 |  | 0.25 |
|  | Junior college  i | | -0.04 | -0.42^*^ |  | 0.12 |  | -0.21* | 0.02 |  | 0.10 |
|  | College  i | | -0.17 | -0.05 |  | -0.00 |  | -0.09 | -0.02 |  | -0.24 |
| *Reasons to choose the current workplace*  l | | | |  |  |  |  |  |  |  |  |
|  | Wage | | 0.07 | 0.29 |  | -0.20 |  | 0.06 | 0.13 |  | 0.65** |
|  | Training | | 2.00** | 1.06* |  | 1.70** |  | 1.61** | 1.00+ |  | 1.48** |
|  | Working hour or day | | 0.04 | 0.43 |  | 0.50+ |  | -0.07 | -0.06 |  | 0.04 |
| *Facility characteristics*  *v* | | |  |  |  |  |  |  |  |  |  |
|  | Nonprofit^b^ | | - | - |  | 0.06 |  | 0.14 | 0.20 |  | 0.12 |
|  | Scale: Middle | | 0.16 | 0.13 |  | - |  | -0.22 | -0.11 |  | -0.08 |
|  | Scale: Large | | 0.59** | 0.50** |  | - |  | -0.21 | -0.19 |  | -0.15 |
| *Turnover opportunity* | | |  |  |  |  |  |  |  |  |  |
|  | Unemployment rate | | -0.04 | 0.08 |  | 0.01 |  | 0.20+ | -0.01 |  | 0.35** |
|  | Large cityd  j | | -0.34 | -0.29 |  | -0.20 |  | -0.24 | -0.53* |  | 0.12 |
|  | Small and medium city  j | | -0.26 | -0.25 |  | -0.29 |  | -0.17 | -0.18 |  | 0.04 |
|  | Year 2018 | | 0.02 | -0.03 |  | -0.13 |  | -0.02 | -0.38** |  | -0.07 |
|  |  | |  |  |  |  |  |  |  |  |  |
| Pseudo R-squared | | | 0.076 | 0.063 |  | 0.100 |  | 0.093 | 0.114 |  | 0.093 |
| *Notes*. HBC=home-based care. Examined using ordered logit model. The dependent variable consists of 5 categories.  ^+^*p*≤0.1. **p*≤.05. ***p*≤.01. | | | | | | | | | | | |

| **Table S4c. Distribution of Training Score (%)** | | | | | | | | | | | |
| --- | --- | --- | --- | --- | --- | --- | --- | --- | --- | --- | --- |
|  | Residential Care | | Group Home | | | Adult Day Service | |  | HBC |  | Total |
| Score | Women | Men |  | Women |  | Women | Men |  | Women |  |  |
| 0 | 9 | 4 |  | 9 |  | 12 | 8 |  | 7 |  | 9 |
| 1 | 19 | 17 |  | 19 |  | 22 | 17 |  | 18 |  | 19 |
| 2 | 20 | 21 |  | 23 |  | 21 | 20 |  | 21 |  | 21 |
| 3 | 18 | 19 |  | 19 |  | 16 | 17 |  | 18 |  | 18 |
| 4 | 13 | 12 |  | 11 |  | 10 | 12 |  | 12 |  | 12 |
| 5 | 7 | 11 |  | 7 |  | 7 | 10 |  | 9 |  | 8 |
| 6 | 13 | 16 |  | 12 |  | 12 | 16 |  | 16 |  | 14 |
| *Notes.* HBC=home-based care. Training score is composed of 6 items (off-the-job training, on-the-job training, case studies, task allocations that match workers’ care ability, a care ability evaluation system, payment according to care ability or qualifications or both). | | | | | | | | | | | |

| **Table S5. Descriptive Statistics for Male Group Home and Home-Based Care Workers** | | | | | | | | |
| --- | --- | --- | --- | --- | --- | --- | --- | --- |
|  | | |  |  |  | Group Home |  | HBC |
|  |  |  | *N* | |  | 303 |  | 292 |
| Dependent variable | | | |  |  |  |  |  |
|  | Turnover intention | | |  |  |  |  |  |
|  |  | Stay | | Yes |  | 55% |  | 66% |
|  |  | Switch | | Yes |  | 14% |  | 8% |
|  |  | Leave | | Yes |  | 7% |  | 5% |
|  |  | Not sure | | Yes |  | 24% |  | 22% |
| Independent variables | | | |  |  |  |  |  |
|  | *Job characteristics* | | |  |  |  |  |  |
|  |  | Hourly wage (yen) | | Mean |  | 1,162 |  | 1,207 |
|  |  | Training (0-6)^a^ | | Mean |  | 3.4 |  | 3.5 |
|  |  | Reliability coefficients for training | | α |  | 0.705 |  | 0.718 |
|  |  | Flexible shift | | Yes |  | 65% |  | 65% |
|  |  | Support (0-2)^a^ | | Mean |  | 0.80 |  | 1.02 |
|  | *Role-related characteristics* | | |  |  |  |  |  |
|  |  | Tenure | | Mean |  | 6.0 |  | 4.8 |
|  |  | Position | |  |  |  |  |  |
|  |  |  | Manager | Yes |  | 14% |  | 21% |
|  |  |  | Assistant manager | Yes |  | 37% |  | 13% |
|  |  |  | Other | Yes |  | 50% |  | 65% |
|  |  | Certified Care Worker | | Yes |  | 70% |  | 48% |
|  |  | Non-regular | | Yes |  | 2% |  | 4% |
|  | *Personal characteristics* | | |  |  |  |  |  |
|  |  | Age | | Mean |  | 39 |  | 40 |
|  |  | Marriage | | Yes |  | 42% |  | 45% |
|  |  | Breadwinner | | Yes |  | 59% |  | 65% |
|  |  | Education | |  |  |  |  |  |
|  |  |  | High school | Yes |  | 54% |  | 58% |
|  |  |  | Junior college | Yes |  | 15% |  | 10% |
|  |  |  | College | Yes |  | 32% |  | 32% |
|  | *Reasons to choose the current workplace* | | | | | | | |
|  |  | Wage | | Yes |  | 9% |  | 10% |
|  |  | Training | | Yes |  | 3% |  | 3% |
|  |  | Working hour or day | | Yes |  | 7% |  | 12% |
|  | *Facility characteristics* | | |  |  |  |  |  |
|  |  | Nonprofit | | Yes |  | 40% |  | 17% |
|  |  | Scale^b^ | |  |  |  |  |  |
|  |  |  | Small | Yes |  | - |  | 31% |
|  |  |  | Middle | Yes |  | - |  | 34% |
|  |  |  | Large | Yes |  | - |  | 35% |
|  | *Turnover opportunity* | | |  |  |  |  |  |
|  |  | Unemployment rate | | Mean |  | 2.5 |  | 2.7 |
|  |  | Population size | |  |  |  |  |  |
|  |  |  | Large city^c^ | Yes |  | 24% |  | 34% |
|  |  |  | Small and medium city | Yes |  | 63% |  | 61% |
|  |  |  | Village | Yes |  | 13% |  | 5% |
|  |  | Year 2018 | | Yes |  | 47% |  | 51% |
| *Notes.* HBC=home-based care. ^a^ Range. ^b^ Number of employees per establishment. middle (10-19), large (20-). Since the numbers of group home employees were small, the scale was omitted. ^C^ Tokyo 23 wards and ordinance-designated city. | | | | | | | | |

| **Table S6. Descriptive Statistics** | | | | | | | | | |
| --- | --- | --- | --- | --- | --- | --- | --- | --- | --- |
|  | | |  | | Total | RC | GH | ADS | HBC |
|  |  |  | *N* | | 7,311 | 2,325 | 1,122 | 2,388 | 1,476 |
|  |  |  | (%) | | 100% | 41% | 73% | 73% | 27% |
| Dependent variable | | | |  |  |  |  |  |  |
|  | Turnover intention | | |  |  |  |  |  |  |
|  |  | Stay | | Yes | 55% | 49% | 53% | 56% | 63% |
|  |  | Switch | | Yes | 13% | 15% | 14% | 12% | 9% |
|  |  | Leave | | Yes | 8% | 9% | 9% | 7% | 6% |
|  |  | Not sure | | Yes | 25% | 27% | 24% | 25% | 23% |
| Independent variables | | | |  |  |  |  |  |  |
|  | *Job characteristics* | | |  |  |  |  |  |  |
|  |  | Hourly wage (yen) | | Mean | 1,128 | 1,199 | 1,123 | 1,061 | 1,128 |
|  |  | Training (0-6)^a^ | | Mean | 2.9 | 2.9 | 2.9 | 2.7 | 3.1 |
|  |  | Reliability coefficients for training | | α | 0.716 | 0.709 | 0.707 | 0.706 | 0.739 |
|  |  | Flexible shift | | Yes | 57% | 55% | 62% | 54% | 58% |
|  |  | Support (0-2)^a^ | | Mean | 0.71 | 0.67 | 0.69 | 0.70 | 0.81 |
|  | *Role-related characteristics* | | |  |  |  |  |  |  |
|  |  | Tenure | | Mean | 6.9 | 8.1 | 6.8 | 6.2 | 6.4 |
|  |  | Duty position | |  |  |  |  |  |  |
|  |  |  | Manager | Yes | 8% | 4% | 11% | 7% | 16% |
|  |  |  | Assistant manager | Yes | 30% | 47% | 33% | 22% | 14% |
|  |  |  | Other | Yes | 61% | 49% | 56% | 71% | 70% |
|  |  | Certified Care Worker | | Yes | 71% | 81% | 73% | 66% | 61% |
|  |  | Non-regular | | Yes | 7% | 5% | 5% | 8% | 10% |
|  | *Personal characteristics* | | |  |  |  |  |  |  |
|  |  | Age | | Mean | 42 | 39 | 44 | 42 | 46 |
|  |  | Marriage | | Yes | 50% | 48% | 43% | 55% | 51% |
|  |  | Breadwinner | | Yes | 46% | 50% | 49% | 41% | 46% |
|  |  | Education | |  |  |  |  |  |  |
|  |  |  | High school | Yes | 63% | 60% | 66% | 61% | 69% |
|  |  |  | Junior college | Yes | 21% | 22% | 19% | 24% | 19% |
|  |  |  | College | Yes | 15% | 18% | 15% | 15% | 12% |
|  | *Reasons to choose the current workplace* | | | | |  |  |  |  |
|  |  | Wage | | Yes | 9% | 10% | 7% | 8% | 11% |
|  |  | Training | | Yes | 2% | 2% | 2% | 2% | 2% |
|  |  | Working hour or day | | Yes | 16% | 7% | 8% | 27% | 19% |
|  | *Facility characteristics* | | |  |  |  |  |  |  |
|  |  | Nonprofit^b^ | | Yes | 62% | 100% | 43% | 52% | 34% |
|  |  | Scale^c^ | |  |  |  |  |  |  |
|  |  |  | Small | Yes | 32% | 1% | 100% | 30% | 35% |
|  |  |  | Middle | Yes | 20% | 8% | 0% | 33% | 31% |
|  |  |  | Large | Yes | 48% | 91% | 0% | 38% | 34% |
|  | *Turnover opportunity* | | |  |  |  |  |  |  |
|  |  | Unemployment rate | | Mean | 2.5 | 2.4 | 2.5 | 2.5 | 2.6 |
|  |  | Population size | |  |  |  |  |  |  |
|  |  |  | Large city | Yes | 18% | 15% | 19% | 17% | 26% |
|  |  |  | Small and medium city | Yes | 65% | 66% | 64% | 67% | 59% |
|  |  |  | Village | Yes | 17% | 19% | 17% | 15% | 14% |
|  |  | Year 2018 | | Yes | 48% | 50% | 44% | 49% | 46% |
| *Notes.* GH=Group Home. ADS=Adult Day Service. HBC=Home-Based Care. The sample includes both males and females. RC=Residential Care.  ^a^ Range.  ^b^ In Japan, only nonprofit entities are allowed to supply residential care.  ^c^ Number of employees per establishment: small (-9), middle (10-19), large (20-). | | | | | | | | | |

| **Table S7a. Multinomial Logistic Regression Results for Turnover intention for Residential Care** | | | | | | | | |
| --- | --- | --- | --- | --- | --- | --- | --- | --- |
|  | | Women | | |  | Men | | |
|  |  | Switch | Leave | Not Sure |  | Switch | Leave | Not Sure |
| *Job characteristics* | |  |  |  |  |  |  |  |
|  | Hourly wage (logged) | -1.18** | -1.13* | -0.54 |  | -1.36** | -1.89** | -0.96* |
|  | Training | -0.12* | -0.24** | -0.04 |  | -0.11 | -0.15 | -0.12* |
|  | Flexible shift | -0.03 | -0.33 | -0.43** |  | -0.48* | -0.57* | -.0.15 |
|  | Support | -0.36** | -0.24 | -0.29* |  | -0.39* | -0.17 | -0.19 |
| *Role-related characteristics* | |  |  |  |  |  |  |  |
|  | Tenure (logged) | -0.02 | 0.35* | 0.05 |  | 0.09 | -0.04 | -0.03 |
|  | Manager | 0.43 | 0.10 | 0.18 |  | -0.11 | -1.20 | -0.43 |
|  | Assistant managert | 0.13 | -0.53* | 0.08 |  | -0.10 | -0.04 | -0.07 |
|  | Certified Care Worker | 0.20 | -0.08 | 0.27 |  | -0.05 | -0.51 | 0.12 |
|  | Non-regular | -0.51 | -0.26 | 0.26 |  | -1.30 | -0.97 | 0.29 |
| *Personal characteristics* | |  |  |  |  |  |  |  |
|  | Age (logged) | -1.39** | -1.14* | -1.17** |  | -0.67 | 0.06 | -0.39 |
|  | Marriage | -0.40* | -0.62* | 0.25 |  | -0.24+ | -0.61* | -0.49** |
|  | Breadwinner | 0.33+ | 0.03 | 0.36** |  | 0.29 | 0.52 | 0.01 |
|  | Junior college | 0.13 | -0.25 | 0.01 |  | 0.07 | 0.14 | 0.03 |
|  | College | 0.82** | 0.57** | 0.36 |  | 0.12 | -1.38** | -0.23 |
| *Reasons to choose the current workplace* | |  |  |  |  |  |  |  |
|  | Wage | -0.27 | 0.24 | -0.19 |  | -0.30 | -0.23 | -0.04 |
|  | Training | -1.20* | -0.61 | -1.01 |  | 0.54 | -0.09 | -0.91 |
|  | Working hour or day | 0.02 | -0.04 | -0.34 |  | -0.86+ | -1.75+ | -0.46 |
| *Facility characteristics* | |  |  |  |  |  |  |  |
|  | Nonprofit | - | - | - |  | - | - | - |
|  | Scale: middle^a^ | 0.22 | -0.36 | 0.08 |  | -0.19 | 0.27 | 0.17 |
|  | Scale: large ^a^ | 0.07 | 0.11 | -0.04 |  | 0.00 | -0.03 | 0.48* |
| *Turnover opportunity* | |  |  |  |  |  |  |  |
|  | Unemployment rate | 0.02 | -0.03 | 0.03 |  | 0.16 | 0.02 | -0.10 |
|  | Large city^c^ | 0.37 | -0.30 | -0.29 |  | -0.24 | 0.28 | 0.18 |
|  | Small and medium city | 0.17 | -0.35 | 0.08 |  | -0.22 | -0.03 | -0.18 |
|  | Year 2018 | -0.13 | -0.14 | 0.05 |  | 0.38 | -0.13 | -0.08 |
|  |  |  |  |  |  |  |  |  |
| n |  | 1376 | | |  | 949 | | |
| Pseudo R-squared | | 0.067 | | |  | 0.067 | | |
| *Notes.* The reference category of the dependent variable is "stay." In account for the possible correlation of variables within prefecture, we used the Huber-White sandwich estimator clustered by prefecture.  ^a^ Number of employees per establishment: middle (20-49), large (50-).  ^b^ Tokyo 23 wards and ordinance-designated city.  ^+^*p*≤0.1. **p*≤.05. ***p*≤.01. | | | | | | | | |

| **Table S7b. Multinomial Logistic Regression Results for Turnover intention for Group Home** | | | | | | | | |
| --- | --- | --- | --- | --- | --- | --- | --- | --- |
|  | | Women | | |  | Men | | |
|  |  | Switch | Leave | Not Sure |  | Switch | Leave | Not Sure |
| *Job characteristics* | |  |  |  |  |  |  |  |
|  | Hourly wage (logged) | -0.81 | -1.02 | -0.19 |  | -1.71^+^ | 1.72* | -0.76 |
|  | Training | -0.34** | -0.23** | -0.16* |  | -0.62** | -0.05 | -0.10 |
|  | Flexible shift | -0.15 | -0.12 | -0.35 |  | 0.62 | -0.66 | -.0.33 |
|  | Support | -0.27+ | -0.28 | -0.51** |  | -0.48 | -0.32 | -0.30 |
| *Role-related characteristics* | |  |  |  |  |  |  |  |
|  | Tenure (logged) | -0.08 | 0.29+ | 0.18 |  | -0.14 | 0.29 | -0.26 |
|  | Manager | -0.12 | 0.03 | -0.43 |  | 0.50 | 0.64 | 0.55 |
|  | Assistant manager | 0.12 | 0.14 | 0.04 |  | 0.57 | 0.59 | 0.05 |
|  | Certified Care Worker | 0.36 | 0.25 | -0.04 |  | 0.84^+^ | -0.58 | -0.14 |
|  | Non-regular | 0.37 | 0.78 | -0.26 |  | -15.36** | -14.53^**^ | -0.92 |
| *Personal characteristics* | |  |  |  |  |  |  |  |
|  | Age (logged) | -1.99** | -2.12** | -1.90** |  | -0.20 | -1.34 | -1.70** |
|  | Marriage | 0.30 | 0.14 | 0.03 |  | 0.78 | -0.11 | 0.16 |
|  | Breadwinner | 0.87** | 0.51+ | 0.52* |  | -0.36 | 0.29 | 0.15 |
|  | Junior college | 0.24 | 0.25 | 0.04 |  | 0.05 | -14.06^**^ | 0.22 |
|  | College | 0.33 | 0.24 | -0.25 |  | 0.31 | 0.37 | 0.44 |
| *Reasons to choose the current workplace* | |  |  |  |  |  |  |  |
|  | Wage | 0.13 | -1.51 | -0.18 |  | -1.05 | 0.03 | 0.19 |
|  | Training | -0.04 | 0.79 | -1.33 |  | 0.56 | -14.55^**^ | 0.41 |
|  | Working hour or day | -1.30* | -1.05* | -0.40 |  | 0.97 | -14.06 | -0.10 |
| *Facility characteristics* | |  |  |  |  |  |  |  |
|  | Nonprofit | 0.15 | -0.09 | -0.02 |  | 0.21 | -0.17 | -0.51+ |
|  | Scale: middle^a^ | - | - | - |  | - | - | - |
|  | Scale: large ^a^ | - | - | - |  | - | - | - |
| *Turnover opportunity* | |  |  |  |  |  |  |  |
|  | Unemployment rate | 0.03 | 0.25 | -0.08 |  | 1.13** | -0.73 | 0.12 |
|  | Large city^c^ | 0.44 | -0.20 | 0.56* |  | 0.04 | -1.30 | -0.04 |
|  | Small and medium city | 0.46* | 0.15 | 0.26 |  | -1.67** | -1.59 | -1.06** |
|  | Year 2018 | 0.10 | 0.10 | 0.19 |  | 0.72^+^ | 0.09 | -0.09 |
|  |  |  |  |  |  |  |  |  |
| n |  | 819 | | |  | 303 | | |
| Pseudo R-squared | | 0.090 | | |  | 0.187 | | |
| *Notes.* The reference category of the dependent variable is "stay." In account for the possible correlation of variables within prefecture, we used the Huber-White sandwich estimator clustered by prefecture.  ^a^ Number of employees per establishment.: middle (10-19), large (20-)  ^b^ Tokyo 23 wards and ordinance-designated city.  ^+^*p*≤0.1. **p*≤.05. ***p*≤.01. | | | | | | | | |

| **Table S7c. Multinomial Logistic Regression Results for Turnover intention for Adult Day Service** | | | | | | | | |
| --- | --- | --- | --- | --- | --- | --- | --- | --- |
|  | | Women | | |  | Men | | |
|  |  | Switch | Leave | Not Sure |  | Switch | Leave | Not Sure |
| *Job characteristics* | |  |  |  |  |  |  |  |
|  | Hourly wage (logged) | -2.35** | -0.88 | -1.20** |  | -2.80** | -3.48** | -2.00** |
|  | Training | -0.14* | 0.07 | -1.77** |  | -0.15 | -0.25* | -0.24** |
|  | Flexible shift | -0.40+ | -0.28 | -0.04 |  | -0.39 | 0.06 | -.0.01 |
|  | Support | -0.14 | -0.74** | -0.28** |  | -0.39+ | -0.48* | -0.02 |
| *Role-related characteristics* | |  |  |  |  |  |  |  |
|  | Tenure (logged) | -0.01 | 0.10 | 0.00 |  | -0.03 | 0.52* | 0.19+ |
|  | Manager | -0.96 | 0.51 | -0.11 |  | 0.15 | 1.23* | 0.50 |
|  | Assistant manager | 0.38+ | -0.04 | -0.22 |  | 0.35 | 0.28 | 0.39+ |
|  | Certified Care Worker | 0.32 | 0.42 | 0.26* |  | 0.09 | -0.37 | -0.31 |
|  | Non-regular | -0.30 | -0.19 | 0.26 |  | 0.34 | 0.63 | -0.10 |
| *Personal characteristics* | |  |  |  |  |  |  |  |
|  | Age (logged) | -1.47** | -0.52 | -0.93** |  | -1.05** | -0.73 | -0.50 |
|  | Marriage | 0.05 | -0.24 | -0.03 |  | 0.49 | -0.05 | -0.28 |
|  | Breadwinner | 0.65** | 0.26 | 0.43* |  | -0.49 | 0.27 | -0.23 |
|  | Junior college | -0.10 | -0.05 | -0.05 |  | -0.21 | -0.25 | 0.34 |
|  | College | 0.45* | 0.33 | 0.28 |  | -0.09 | 0.20 | -0.14 |
| *Reasons to choose the current workplace* | |  |  |  |  |  |  |  |
|  | Wage | 0.00 | -0.53 | -0.44* |  | 0.30 | 0.98 | 0.21 |
|  | Training | -0.27 | -0.47 | -0.56 |  | -0.57 | -12.8** | -0.51 |
|  | Working hour or day | -0.26 | -0.16 | -0.09 |  | -0.80+ | -0.47 | -0.19 |
| *Facility characteristics* | |  |  |  |  |  |  |  |
|  | Nonprofit | 0.00 | 0.35 | 0.01 |  | 0.22 | -0.46 | 0.11 |
|  | Scale: middle^a^ | 0.16 | -0.13 | 0.37* |  | 0.75+ | -0.21 | 0.48* |
|  | Scale: large ^a^ | 0.17 | -0.10 | 0.45** |  | 0.72+ | -0.33 | 0.13 |
| *Turnover opportunity* | |  |  |  |  |  |  |  |
|  | Unemployment rate | 0.04 | -0.15 | -0.10 |  | 0.41 | 0.13 | -0.21 |
|  | Large city^c^ | -0.25 | -0.00 | 0.14 |  | 1.67** | 0.55 | 0.12 |
|  | Small and medium city | -0.09 | 0.33 | 0.10 |  | 1.18* | 0.11 | -0.10 |
|  | Year 2018 | 0.08 | -0.02 | 0.18 |  | 0.56^+^ | 0.64^+^ | 0.13 |
|  |  |  |  |  |  |  |  |  |
| n |  | 1753 | | |  | 635 | | |
| Pseudo R-squared | | 0.065 | | |  | 0.110 | | |
| *Notes.* The reference category of the dependent variable is "stay." In account for the possible correlation of variables within prefecture, we used the Huber-White sandwich estimator clustered by prefecture.  ^a^ Number of employees per establishment.: middle (10-19), large (20-)  ^b^ Tokyo 23 wards and ordinance-designated city.  ^+^*p*≤0.1. **p*≤.05. ***p*≤.01. | | | | | | | | |

| **Table S7d. Multinomial Logistic Regression Results for Turnover intention for Home-Based Care** | | | | | | | | |
| --- | --- | --- | --- | --- | --- | --- | --- | --- |
|  | | Women | | |  | Men | | |
|  |  | Switch | Leave | Not Sure |  | Switch | Leave | Not Sure |
| *Job characteristics* | |  |  |  |  |  |  |  |
|  | Hourly wage (logged) | -0.77 | -2.46** | -1.11** |  | -2.37 | -0.18 | 0.62 |
|  | Training | -0.17* | -0.06 | -0.06 |  | -0.53^**^ | -0.05 | -0.35** |
|  | Flexible shift | -0.70* | -1.09** | -0.74** |  | -1.24^*^ | -0.26 | 0.16 |
|  | Support | -0.30+ | -0.43+ | -0.29+ |  | 0.23 | 0.37 | 0.15 |
| *Role-related characteristics* | |  |  |  |  |  |  |  |
|  | Tenure (logged) | 0.07 | 0.18 | 0.02 |  | 0.04 | 0.34 | -0.06 |
|  | Manager | -0.13 | -0.11 | 0.36+ |  | -1.28 | 1.62^+^ | -0.63+ |
|  | Assistant manager | -0.12 | 0.34 | 0.36* |  | 1.05 | 1.25 | 0.16 |
|  | Certified Care Worker | 0.24 | 0.11 | -0.07 |  | 0.65 | 1.03 | 1.09** |
|  | Non-regular | -0.26 | -0.51 | 0.04 |  | 0.48 | 2.45 | 0.94 |
| *Personal characteristics* | |  |  |  |  |  |  |  |
|  | Age (logged) | -0.87* | 0.17 | -0.78** |  | -0.74 | 1.20 | -0.67 |
|  | Marriage | -0.87** | -0.73+ | -0.10 |  | 0.07 | -0.99 | -0.03 |
|  | Breadwinner | -0.31 | -0.24 | 0.22 |  | -1.83^**^ | -1.79^**^ | -0.49 |
|  | Junior college | 0.06 | -0.21 | 0.19 |  | 1.46^*^ | 0.85 | -0.25 |
|  | College | 0.18 | -0.57 | -0.47 |  | 0.14 | -0.05 | -0.40 |
| *Reasons to choose the current workplace* | |  |  |  |  |  |  |  |
|  | Wage | 0.38 | 0.07 | -0.65** |  | -0.40 | -0.17 | -1.42* |
|  | Training | 0.10 | -12.7** | -1.33 |  | -12.34^**^ | -14.99^**^ | 0.80 |
|  | Working hour or day | -0.45 | -0.50 | -1.72 |  | -0.98 | 0.57 | -0.16 |
| *Facility characteristics* | |  |  |  |  |  |  |  |
|  | Nonprofit | -0.07 | -0.21 | 0.02 |  | 0.88 | 0.42 | -0.74 |
|  | Scale: middle^a^ | 0.29 | 0.52+ | -0.03 |  | -1.02 | 0.32 | 0.02 |
|  | Scale: large ^a^ | 0.24 | 0.03 | 0.13 |  | -0.46 | 0.26 | 0.24 |
| *Turnover opportunity* | |  |  |  |  |  |  |  |
|  | Unemployment rate | -0.06 | -0.49+ | 0.08 |  | 0.41 | 1.23^+^ | -0.25 |
|  | Large city^c^ | -0.22 | 0.16 | 0.39 |  | 13.73^**^ | -2.57^*^ | 15.16** |
|  | Small and medium city | -0.13 | 0.06 | 0.21 |  | 13.48^**^ | -2.37^*^ | 14.71** |
|  | Year 2018 | 0.61+ | -0.12 | 0.29* |  | 0.73 | 1.02 | 0.18 |
|  |  |  |  |  |  |  |  |  |
| n |  | 1184 | | |  | 292 | | |
| Pseudo R-squared | | 0.089 | | |  | 0.210 | | |
| *Notes.* The reference category of the dependent variable is "stay." In account for the possible correlation of variables within prefecture, we used the Huber-White sandwich estimator clustered by prefecture.  ^a^  Number of employees per establishment.: middle (10-19), large (20-)  ^b^ Tokyo 23 wards and ordinance-designated city.  ^+^*p*≤0.1. **p*≤.05. ***p*≤.01. | | | | | | | | |

| **Table S8. Multinomial Logistic Regression Results for Turnover intention where "Leavers" are Separated** | | | | | | | | | | | | | | | | | |
| --- | --- | --- | --- | --- | --- | --- | --- | --- | --- | --- | --- | --- | --- | --- | --- | --- | --- |
|  | Residential Care | | | | |  | Group Home | |  | Adult Day Service | | | | |  | Home Based Care | |
|  | Women | |  | Men | |  | Women | |  | Women | |  | Men | |  | Women | |
|  | Non-health care | Stop working |  | Non-health care | Stop working |  | Non-health care | Stop working |  | Non-health care | Stop working |  | Non-health care | Stop working |  | Non-health care | Stop working |
| Hourly wage (logged) | -1.04 | -1.43+ |  | -1.78** | -2.62* |  | -0.53 | -3.11** |  | -0.86 | -1.17 |  | -3.73** | -2.93^+^ |  | -2.41** | -2.49** |
|  | (0.65) | (0.84) |  | (0.65) | (1.27) |  | (0.75) | (1.20) |  | (0.84) | (0.76) |  | (0.91) | (1.56) |  | (0.84) | (0.76) |
| Wald test statistic^a^ | 0.14 | |  | 0.44 | |  | 3.21+ | |  | 0.08 | |  | 0.20 | |  | 0.01 | |
|  |  |  |  |  |  |  |  |  |  |  |  |  |  |  |  |  |  |
| Training | -0.27** | -0.17 |  | -0.10 | -0.31 |  | -0.19* | -0.42* |  | 0.03 | 0.18 |  | -0.16 | -0.58* |  | -0.05 | -0.10 |
|  | (0.10) | (0.15) |  | (0.09) | (0.23) |  | (0.10) | (0.21) |  | (0.09) | (0.11) |  | (0.12) | (0.27) |  | (0.12) | (0.17) |
| Wald test statistic^a^ | 0.35 | |  | 0.80 | |  | 1.25 | |  | 0.93 | |  | 1.99 | |  | 0.07 | |
| *Notes.* The reference category of the dependent variable is "stay." Only the coefficients of hourly wage and training are shown. The results of "switch" and “not sure” are omitted. Standard errors in parenthesis. Leavers compose of individuals who want to switch to a non-health care setting and those who want to leave the labor force altogether.  ^a^ The equalities of the coefficients of “non-health care” and “stop working” are checked by the Wald test. +p≤0.1. *p≤.05. **p≤.01. | | | | | | | | | | | | | | | | | |
